# Statistical Approaches in the Studies Assessing Associations between Human Milk Immune Composition and Allergic Diseases: A Scoping Review

**DOI:** 10.3390/nu11102416

**Published:** 2019-10-10

**Authors:** Oleg Blyuss, Ka Yan Cheung, Jessica Chen, Callum Parr, Loukia Petrou, Alina Komarova, Maria Kokina, Polina Luzan, Egor Pasko, Alina Eremeeva, Dmitrii Peshko, Vladimir I. Eliseev, Sindre Andre Pedersen, Meghan B. Azad, Kirsi M. Jarvinen, Diego G. Peroni, Valerie Verhasselt, Robert J. Boyle, John O. Warner, Melanie R. Simpson, Daniel Munblit

**Affiliations:** 1 Wolfson Institute of Preventive Medicine, Queen Mary University of London, London EC1M 6BQ, UK; o.blyuss@qmul.ac.uk; 2 Department of Paediatrics and Paediatric Infectious Diseases, Institute of Child’s Health, Sechenov First Moscow State Medical University (Sechenov University), 123337 Moscow, Russia; ya.alinaowl@yandex.ru (A.K.); mary_stars@mail.ru (M.K.); polinkaluzan@yandex.ru (P.L.); xfanchio@gmail.com (E.P.); alinaeremeeva@yandex.ru (A.E.); dmitrii.peshko@icloud.com (D.P.); 3 Department of Paediatrics, Imperial College London, London W2 1PG, UK; ka.cheung15@imperial.ac.uk (K.Y.C.); jessica.chen16@imperial.ac.uk (J.C.); callum.parr16@imperial.ac.uk (C.P.); loukia.petrou15@imperial.ac.uk (L.P.); r.boyle@nhs.net (R.J.B.); j.o.warner@imperial.ac.uk (J.O.W.); 4 N. Polyakov Institute of Geotechnical Mechanics on the NAS of Ukraine, 49005 Dnipro, Ukraine; vladimir.eliseev@yahoo.com; 5 Library Section for Medicine and Health Sciences, NTNU—Norwegian University of Science and Technology, 7030 Trondheim, Norway; sindre.a.pedersen@ntnu.no; 6 Department of Pediatrics and Child Health, Children’s Hospital Research Institute of Manitoba, University of Manitoba, Winnipeg, MB R3E 3P4, Canada; meghan.azad@umanitoba.ca; 7 Division of Pediatric Allergy and Immunology & Center for Food Allergy, University of Rochester School of Medicine and Dentistry, Rochester, New York, NY 14642, USA; kirsi_jarvinen-seppo@urmc.rochester.edu; 8 Department of Clinical and Experimental Medicine, Section of Paediatrics, University of Pisa, 56126 Pisa, Italy; diego.peroni@unipi.it; 9 School of Molecular Sciences, University of Western Australia, Perth, WA 6009, Australia; valerie.verhasselt@uwa.edu.au; 10 inVIVO Planetary Health, Group of the Worldwide Universities Network (WUN), West New York, NJ 10704, USA; 11 National Institute for Health Research, Collaboration for Leadership in Applied Health Research and Care for NW London, London SW10 9NH, UK; 12 Department of Public Health and General Practice, NTNU – Norwegian University of Science and Technology, 7030 Trondheim, Norway; melanie.simpson@ntnu.no; 13 Clinic of Laboratory Medicine, St Olavs Hospital, 7030 Trondheim, Norway; 14 Solov’ev Research and Clinical Center for Neuropsychiatry, 115419 Moscow, Russia

**Keywords:** breast milk, colostrum, immune composition, human milk, immune markers, allergy, longitudinal algorithms, methodology, serial analysis, statistical analysis

## Abstract

A growing number of studies are focusing on the associations between human milk (HM) immunological composition and allergic diseases. This scoping review aims to identify statistical methods applied in the field and highlight pitfalls and unmet needs. A comprehensive literature search in MEDLINE and Embase retrieved 13,607 unique records. Following title/abstract screening, 29 studies met the selection criteria and were included in this review. We found that definitions of colostrum and mature milk varied across the studies. A total of 17 out of 29 (59%) studies collected samples longitudinally, but only 12% of these used serial (longitudinal) analyses. Multivariable analysis was used in 45% of the studies, but statistical approaches to modelling varied largely across the studies. Types of variables included as potential confounding factors differed considerably between models. Discrimination analysis was absent from all studies and only a single study reported classification measures. Outcomes of this scoping review highlight lack of standardization, both in data collection and handling, which remains one of the main challenges in the field. Improved standardization could be obtained by a consensus group of researchers and clinicians that could recommend appropriate methods to be applied in future prospective studies, as well as already existing datasets.

## 1. Introduction

The prevalence of allergic disease has been rising worldwide in the past decades [1]. This pattern is particularly evident in westernised and urbanised countries [2], but recent data suggest that it is also an emerging problem in the developing world [3]. “The Hygiene Hypothesis” has been proposed as the main theory to explain the increase in prevalence of allergic disease [4], pointing at a reduced repertoire of specific immunological responses to different organisms due to reduced stimulation of the immune system by microbial antigens associated with modern living.

Human breast milk is a known contributor to neonatal infection prevention and recent data show that it may also reduce the risk of non-communicable diseases. It is possible that the pre-biotic properties of human milk (HM) contribute to the latter by enhancing the establishment of a disease impairing gut microbiome [5]. A systematic review by Lodge and co-authors suggested protection against asthma development, with weak evidence of association between breastfeeding and eczema [6] with heterogeneity in methodology reported across studies. Some experts have suggested that conflicting results may be linked to variation in HM composition [7,8,9]. Furthermore, some biologically active components in HM are suspected to be modified by interventions in the maternal diet and lifestyle during late pregnancy and lactation [9,10], subsequently influencing infant health outcomes [11,12].

Many studies assessing HM composition have focused on diverse allergic outcomes such as allergic sensitisation, eczema, food allergy and/or wheeze/asthma development in infancy and there is a need for systematic analysis of evidence for each condition independently [13]. Although several systematic reviews were recently published on this topic [14,15,16], appropriate meta-analysis of the data was not possible due to methodological inconsistencies between the studies.

It has been recently demonstrated that even when the same dataset is analysed independently by different research groups, methodological approaches and outcomes of the statistical analysis vary significantly [17]. In the experimental work, 29 research teams, involving 61 analysts, used a wide range of statistical models, and 21 unique combinations of covariates, which led to the estimated effect size ranging from 0.89 to 2.93 [18]. When study authors discussed approaches and results with members of the represented research groups, no consensus emerged on a single, best approach. This illustrates the importance of a proper justification of the statistical approach selected in a given setting and need for standardisation of statistical analyses.

Serial (longitudinal) samples (e.g., colostrum and mature milk, or longitudinal mature milk samples) are often collected in HM research, however, they are rarely analysed in a serial fashion. The importance of this approach has recently attracted increased attention in other disciplines, most notably oncology. The incorporation of longitudinal algorithms in a cancer-screening program resulted in a substantial increase in the predictive value of risk models [19].

This scoping review provides a critical appraisal of statistical approaches used in studies investigating associations between HM immunological composition and allergic disease outcomes, with an emphasis on serial analysis.

## 2. Materials and Methods

Our methods were based on Preferred Reporting Items for Systematic reviews and Meta-Analyses extension for Scoping Reviews (PRISMA-ScR). A protocol outlining the process was prepared prior to this scoping review. The protocol has not been registered at PROSPERO as it does not currently accept registrations for scoping reviews, literature reviews or mapping reviews. This work, however, is complementary to the ongoing series of systematic reviews looking at available evidence on HM immunological composition and health outcomes and registered at PROSPERO (PROSPERO 2019 CRD42019126893 and PROSPERO 2019 CRD42019126894).

### 2.1. Search Strategy

In the process of conducting the systematic reviews mentioned in the previous section, an extensive electronic search was performed in the bibliographic databases MEDLINE and Embase using both thesaurus and free-text terms to identify bibliographic records involving HM and immunological composition, such as cytokines, chemokines and immunoglobulins (see Supplementary Material for detailed description of the literature search). The literature search was last updated on 11 December 2018. All records were imported into EndNote reference manager and all duplicates removed from the library. For the purpose of this scoping review, we employed the EndNote library to identify all records examining associations between HM immunological composition and allergic diseases.

### 2.2. Eligibility Criteria and Selection of Articles

For the purpose of this research, studies of all designs were included if the following criteria were met: (1) reported original data; (2) clinical study of mother–infant dyads; (3) the study had an epidemiological design: observational studies (i.e., pregnancy cohort study, birth cohort study, human prospective study or randomized controlled trial), during pregnancy or lactation and interventional studies; (4) included a quantitative assessment of HM immunological constituents (cytokines, chemokines and/or immunoglobulins); and (5) investigated associations between HM immunological constituents and at least one allergic disease, or allergic sensitization, in a child with follow-up from 6 months to 18 years.

Reviews, conference abstracts, editorials, letters to the editor, case reports and/or case series were excluded from the analysis. Specific immunoglobulins in HM was not part of this research.

To reduce potential selection bias, eight independent investigators (C.P., J.C., L.P., K.C., P.L., E.P., A.K. and M.K.) reviewed all titles and abstracts identified by the search for inclusion. Four researchers (C.P., J.C., L.P. and K.C.) independently reviewed full texts of all publications selected for data extraction. Any disagreements were resolved through discussion involving additional reviewers (O.B. and D.M.) until consensus was reached.

### 2.3. Data Extraction

The data from each study were extracted in duplicate, tabulated, and included the following fields: author(s) and year of publication; descriptive information concerning the study design; country and setting; number and timing of sample collection; details of statistical analysis; adjustment for potential confounders; outcome definition; age of outcome assessment.

## 3. Results

### 3.1. Synthesis

**Figure 1:**
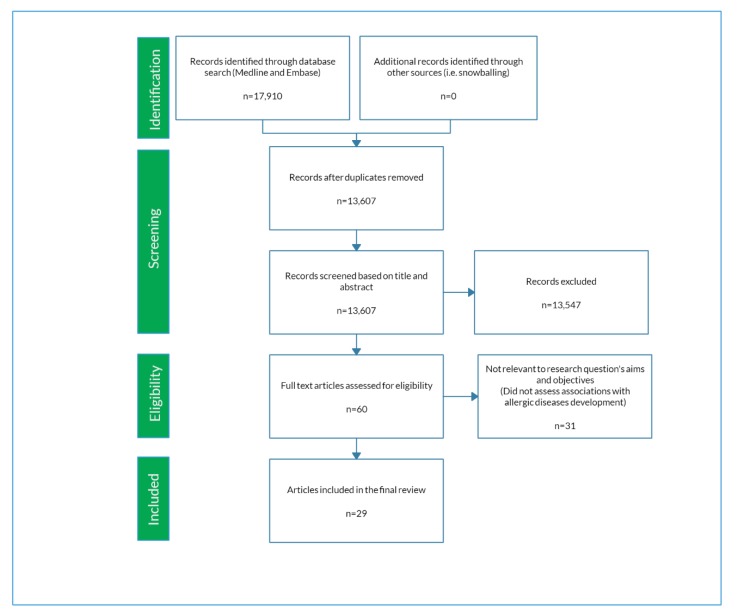
Preferred Reporting Items for Systematic reviews and Meta-Analyses (PRISMA) chart describing study selection process.

Based on the search strategy, a total of 13,607 unique records were identified and screened for eligibility (Figure 1). A total of 29 studies met our stated eligibility and selection of full-text articles criteria and we proceeded to full-text assessment and data extraction. All included studies were published between 1986 and 2018.

### 3.2. Sample Collection and Analysis

Authors reported colostrum/early milk collection within the first 10 days of life [20,21,22,23,24,25,26,27,28,29,30,31,32,33,34], and mature milk between seven days [35,36] and nine months [37] postpartum, with most of the studies collecting one-month milk. The number of immunological markers measured varied between one [25,38,39,40,41] and 50 [30] in different studies, with a median of four across the studies.

A total of 17 out of 29 studies collected samples at two [21,23,24,25,26,27,28,29,31,32,33,34,42,43] or more [25,37,38,41] timepoints. Only two [31,38] of these studies reported serial data analysis, and a single study [44] used a dimensionality reduction approach as part of its statistical analysis (Table 1).

### 3.3. Statistical Methods and Confounders

In total, 13 out of 29 studies analysed data using univariable analysis [20,21,23,26,32,34,37,38,39,41,42,43,45]. The most commonly used approach included the Mann–Whitney [21,23,27,31,33,34,39,42,43,45] and *t*-test [22,23,25,27,37,45]. Multivariable analysis was used in 16 studies [22,24,25,27,28,29,30,31,33,35,36,40,44,46,47,48], with logistic regression being the preferred approach to modelling [22,24,27,28,31,40,46,47,48]. Other techniques included Cox regression [30,44], generalized estimating equations (GEE) [35,36], binomial GLmulti [29] and least absolute shrinkage and selection operator (LASSO) [29].

Adjustment for potential confounding factors or use of covariates was reported in 17 studies [22,24,25,27,28,29,30,31,35,36,38,40,43,44,46,47,48], and in all studies since 2013 [27,28,29,30,31,35,36,38,40,43,44,46,47,48], apart from Hogendorf et al. [39]. Maternal atopy [24,28,29,30,31,35,44,46,47,48], child gender [25,30,31,35,44,46,48] and maternal smoking were the most commonly used confounders across the studies (Table 2).

Association between HM immunological markers and allergic outcomes was reported as a difference in means [20,22,25,37,38,45,46] or medians [21,23,27,31,34,39,42,43], odds [22,24,26,27,28,29,31,40,46,47,48], risk [35,36] or hazardous [30,44] ratios. In a single study, the levels of cytokines were divided into tertiles and association between infant sensitization and levels of immunological markers in HM was reported descriptively [32]. No studies reported discrimination analysis (receiver operating characteristic (ROC) curve, area under the curve (AUC)), with a single study using classification measures (sensitivity, specificity) [38]. Approaches to statistical analysis are summarised in Table 3.

## 4. Discussion

Human milk research aims to detect multiple biologically active components and associations with health outcomes. We are still far from fully understanding the complex composition of HM and its potential consequences for infant health. Emerging evidence generated by prospective cohorts may improve our understanding of the composition role of HM in health and disease. To generate plausible results, which later can be used in meta-analyses, it is highly important to apply the most appropriate and effective methodology.

Lack of standardisation is still a major issue in HM research and results from our scoping review highlight this. Among the 29 identified studies examining associations between immune constituents of HM and allergic diseases, we found that even though most studies collected samples longitudinally, very few performed serial analysis. Definitions of colostrum were very broad and often overlapped with mature milk between the studies. Potential confounding factors differed considerably between models and no studies used discrimination analysis, with only one using classification measures.

Three stages in human lactation are normally identified and explained by changes in specific components of the breast milk (e.g., whey proteins, lactose). These compositional changes likely reflect the changing physiological needs of the infant, and can be stratified into colostrum, transitional milk and mature milk. Unfortunately, there is little alignment in the precise definition of the terms mentioned above. According to various sources, colostrum is the special milk secreted in the first 2–3 days [49] or 1–5 days [50] after delivery; transitional milk is usually produced 7–14 [49] or 5–21 [50] days postpartum; and mature milk after two weeks [49] or >21 days [50] post-partum. These discrepancies cause significant heterogeneity in sample collection timing and HM definition between the studies. Improved standardisation will allow for better harmonisation of datasets.

Serial collection is widely used in the field of human milk research. Regrettably, four of 10 studies failed to collect HM samples longitudinally, at different time points. Even several of the recent studies collected HM samples only once [30,35,44].

Out of 17 studies collecting serial samples, only 12% (2/17) performed serial analysis using appropriate methods. For example, Jarvinen et al. used ANOVA for repeated measurements studying relationships between HM IgA and cow’s milk allergy [38], and Morita and co-authors reported associations between Transforming Growth Factor (TGF)β ratio (mature milk/colostrum) and development of eczema in offspring [31]. Longitudinal analysis provides additional insights into the personalized predictive models and some promising approaches were recently successfully adopted in cancer research [51], showing that serial models significantly outperform non-serial in terms of risk prediction. Applying similar approaches in the field of HM research may potentially allow the development of predictive models for the development of a variety of outcomes, including allergic diseases. Current lack of longitudinal analysis is surprising and is possibly linked with the lack of funding, with many studies failing to utilise some of the information collected (i.e., links between sequential sample collections). In an ‘ideal world’ scenario, sample collection should be longitudinal, but this may not always be feasible, particularly in large cohort studies where HM collection and analysis is not the primary focus. With that being said, sample collection limited to a single time point, restricts statistical analysis to the comparison with population characteristics as some individual’s previous information cannot be taken into account. Potential approaches to be used may include mixed models, which are commonly applied to the analysis of repeated measurements [52], methods that analyse trend indices to evaluate the dynamics [51], as well as more sophisticated techniques, including Bayesian changepoint models [53,54,55], and recurrent neural networks [56]—one of the most prominent deep learning techniques utilizing serial measurements. The listed approaches could be used to describe serial patterns in infants, with or without allergic diseases, thus enabling improved discrimination.

A wide selection of statistical methods was applied in the included studies. The spectrum ranged from simple comparison of two groups using the *t*-test and/or Mann–Whitney to regression models and dimensionality reduction approaches. In total, 41% of the studies (12/29) failed to conduct multivariable analysis, which may negatively impact the validity of the reported data. Exclusion of potentially important variables from the analysis may lead to misleading results. Since 2013, almost all studies have reported multivariable analysis use, which is very promising. In agreement with the study by Silberzahn et al. [18], we found that different groups tend to choose very different statistical methods for assessing the relationship between HM immune active molecules and allergic outcomes. While there is always a variety of statistical approaches that could be used in each setting, such diversity makes merging data-pooled estimates problematic. We therefore encourage adopting methods that generate characteristics that allow comparison in potential future meta-analyses such as odds ratio, relative risk and hazard ratio. This would also require standardization of units of concentration and choice of transformation or normalization methods prior to statistical analyses.

Similar trends were found in relation to confounding factors adjustment or use of covariates, with only 59% of the studies (17/29) considering potential confounders, and all the studies published since 2013 providing adjustment. The selection of potential confounders varied significantly between the studies, with maternal allergy, infant gender and maternal smoking being among the most frequently used. Interestingly, alcohol consumption during pregnancy and/or lactation has not been used as a potential confounder in the modelling and is of interest to be collected in the future. Appropriate use of statistical methods, as well as correct adjustment for potential confounding factors, is highly important in yielding valid results and avoiding possible bias. Silberzahn et al. recently demonstrated that application of identical statistical methods may lead to opposing results depending on the selection of confounding factors [18]. Future research in the field should provide adjustment for potential confounding factors, as there are data suggesting maternal and environmental characteristics may significantly influence both HM immune composition and allergic disease [57], however, a systematic review of available evidence is still needed.

In the studies assessing multiple biologically active molecules in a limited number of samples it is always hard to assess whether associations reflect real causal relationships or are just related to multiple hypothesis testing. This is certainly the case in HM research, where associations between the levels of different HM constituents with disease development are tested. There is no reliable way to prevent this and multiple testing correction methods (e.g., Bonferroni, Holm, Benjamini and Hochberg) are normally applied in order to reduce the risks of invalid interpretation. Unfortunately, a very limited number of reviewed studies adjusted the results for multiple testing. This implies that outcomes of some studies may be overstated, as not taking into account the possible impact of multiple simultaneous testing can greatly increase the probability of false positive findings [58]. We highly recommend use of the multiple testing technique in future HM research.

Reviewed studies of HM immune composition have assessed the association between the levels of various constituents and allergic diseases development, but none of them performed discrimination analysis, even though odds/risk/hazard ratios were reported in some cases. Use of discrimination analysis (e.g., ROC curves, AUC), and classification measures (e.g., sensitivity, specificity, positive predictive value (PPV), negative predictive value (NPV)), would determine whether immunologically active molecules in HM may be used as a predictive tool for allergic outcomes development. We stress the importance of not only reporting the effect sizes of the associations, but also corresponding discrimination characteristics, in order to allow direct comparison of models and populations across the studies. We acknowledge, however, that ROC analyses can be informative, but is not necessarily the goal of all HM studies. Identification of HM components association with health outcomes is often needed to understand disease mechanisms.

The strength of our review is its comprehensive scope and attention to statistical analysis detail. The limitations are related to the methodology of the scoping review and include the possibility that the review may have missed some relevant studies due to database selection, exclusion of grey literature from the search and exclusion of studies not published in English. For the purpose of this review the ‘immunological composition’ is limited to cytokines, chemokines and immunoglobulins. We acknowledge that other components (e.g., human milk oligosaccharides, fatty acids and cells) might have immunological functions. Since our scoping review is focused on the approach to data handling and analysis, this limitation is unlikely to influence the results.

## 5. Conclusions

Although some improvements were seen in the past five years, there was a high degree of heterogeneity across the studies regarding the methodology of data collection and analysis. This scoping review assessed statistical approaches used in the field and critically appraised available evidence. We found that there is a lack of agreement in both, data collection (variations in definitions of human milk “maturity”, lack of longitudinal sample collection) and data analysis (lack of multivariable analysis, serial analysis, use of discrimination analysis/classification measures, adjustment for important confounding factors). Better funding could possibly lead to improvements in methodology and longitudinal samples collection.

Lack of standardization is still one of the main challenges in the field, which makes meta-analysis difficult. We call national and international societies focused on human milk research to establish a consensus group, involving clinicians, researchers, methodologists and biostatisticians to produce proposals for standardization which could then be applied to already available datasets as well as future prospective studies.

## Figures and Tables

**Table 1:** Serial sample collection and serial data analysis in the studies assessing associations between human milk (HM) immunological composition and allergic diseases.

Author, Year	Country	Number of Participants	Number of HM Samples Collected	Serial Sample Collection	Number of Immune Markers Measured	Serial Analysis ^1^	Dimension Reduction/ Clustering ^2^
Machtinger 1986 [41]	USA	57	NR^▪^	Yes	1	No	No
Savilahti 1991 [37]	Finland	161	102 C (NR) 204 MM (2,6,9 mo)	Yes	6	No	No
Kalliomaki 1999 [42]	Finland	47	43 C (NR) 38 MM (3 mo)	Yes	2	No	No
Saarinen 1999 [20]	Finland	315	315 C (1–4 d)	No	5	N/A	No
Jarvinen 2000 [38]	Finland	87	NR MM (2w, 1, 3, 6 mo)	Yes	1	Yes	No
Rautava 2002 [45]	Finland	62	NR ^▪▪^ MM (3 mo)	No	2	N/A	No
Bottcher 2003 [21]	Sweden	53	53 C (4 d) 47 MM (1 mo)	Yes	13	No	No
Oddy 2003 [46]	USA	243	243 MM (2 w)	No	4	N/A	No
Savilahti 2005 [22]	Finland	228	228 C (1–4 d)	No	4	N/A	No
Rigotti 2006 [23]	Italy	22	22 C (3 d) 22 MM (1 mo)	Yes	2	No	No
Snijders 2006 [47]	Netherlands	315	315 MM (1 mo)	No	5	N/A	No
Bottcher 2008 [24]	Sweden	109	109 C (<3 d) 109 MM (1 mo)	Yes	7	No	No
Huurre 2008 [32]	Finland	Between 118 and 126	58 С (1 d) 68 (1 mo)	Yes	7	No	No
Prescott 2008 [33]	New Zealand	105	239 MM (7d, 3, 6 mo)	Yes	8	No	No
Tomicic 2010 [34]	Estonia, Sweden	99	99 C (0-4 d) 99 MM (1 mo)	Yes	7	No	No
Pesonen 2011 [25]	Finland	169	169 C (5 d) 286 MM (2, 6 mo)	Yes	1	No	No
Kuitunen 2012 [26]	Finland	364	364 C (0–3 d) 321 MM (3 mo)	Yes	7	No	No
Soto-Ramírez 2012 [36]	United States of America	115	115 MM (1–8 w)	No	13	N/A	No
Hogendorf 2013 [39]	Poland	84	84 MM (NR)	No	1	N/A	No
Ismail 2013 [43]	Australia, Malaysia, UK	79	158 MM (7, 28d)	Yes	3	No	No
Ochiai 2013[27]	Japan	98	98 C (4–5 d) 98 MM (1 mo)	Yes	26	No	No
Orivuori 2013 [48]	Austria, Finland, France, Germany and Switzerland	610	610 MM (2 mo)	No	2	N/A	No
Joseph 2014 [40]	USA	304	304 MM (1 mo)	No	1	N/A	No
Jepsen 2016 [44]	Denmark	223	223 MM (1 mo)	No	14	N/A	Yes
Simpson 2016 [28]	Norway	259	255 MM (10 d) 247 MM (3 mo)	Yes	4	No	No
Soto-Ramírez 2016 [35]	United States of America	115	115 MM (1–8 w)	No	13	N/A	No
Munblit 2017 [29]	UK, Italy, Russia	398	398 C (6 d) 153 MM (4–6 w)	Yes	11	No	No
Morita 2018 [31]	Japan	96	96 C (5 d) 96 MM (1 mo)	Yes	2	Yes	No
Berdi 2019 [30]	France	263	263 C (2–6 d)	No	50	N/A	No

Abbreviations; C, colostrum; D, days; Mo, months; MM, mature milk; NA, not applicable; NR, not reported; W, weeks. ^1^ Serial analysis was considered as positive if any attempts were undertaken to handle data as serial measurements rather than single time-point variables; ^2^ Latent class analysis (LCA), Principal component analysis (PCA), interactions and scanning electron microscopy (SEM) were used in the analysis; ^▪^ an average of 3.5 mature milk samples were obtained per mother; ^▪▪^ 62 mother–infant pairs participated in the study.

**Table 2:** Confounding factors/covariates reported in the reviewed studies, assessing associations between HM immunological composition and allergic diseases.

Confounding Factors	Frequency	Reference
Maternal atopy	11	[24,28,29,30,31,35,38,44,46,47,48]
Child gender	7	[25,30,31,35,44,46,48]
Maternal smoking	6	[25,28,30,35,44,46]
Breastfeeding duration	4	[22,25,43,44]
Maternal age	4	[30,35,44,47]
Number of siblings	4	[25,28,43,47]
Family history of atopy	3	[22,25,35]
Site of collection	3	[29,30,48]
Exposure to other children	2	[44,46]
Maternal educational level	2	[30,46]
Mode of delivery	2	[43,44]
Probiotics	2	[28,47]
Sibling atopy	2	[29,30]
Colostrum collection time/infant age	2	[29,38]
Birth weight	1	[46]
BMI before pregnancy	1	[30]
Breastfeeding by 1 month and Transforming Growth Factor (TGF)β ratio	1	[31]
C-section	1	[30]
Gestational age	1	[46]
Household income	1	[44]
Household pets	1	[43]
Introduction of food during first year of life	1	[48]
Maternal consumption of acetaminophen during pregnancy	1	[35]
Maternal infection	1	[47]
Maternal marital status	1	[35]
Maternal race	1	[35]
Mother’s alcohol use (3^rd^ trimester)	1	[44]
Mother’s antibiotic use (3^rd^ trimester)	1	[44]
Na^+^/K^+^ ratios	1	[24]
Season of birth	1	[35]
Season of breast milk collection	1	[47]
Study treatment	1	[24]
Time interval between births	1	[47]
Vaginal or urinary infections during pregnancy	1	[35]
Yoghurt and antibiotic consumption during pregnancy	1	[43]

**Table 3:** Statistical approaches to data handling, adjustment for potential confounding factors and use of discrimination and classification measures in the studies assessing associations between HM immunological composition and allergic diseases.

Author, Year	Univariable/MultivariableAnalysis	Statistical Method of HM Marker/Outcome Assessment	Confounders ^1^	Association Reporting	Discrimination Analysis ^2^Yes/No	Classification Measures ^3^
Machtinger 1986 [41]	Univariable	Chi-squared, Fisher’s	No	Proportions	No	No
Savilahti 1991 [20]	Univariable	*t*-test	No	Mean differences	No	No
Kalliomaki 1999 [42]	Univariable	Kruskal–Wallis,Mann–Whitney	No	Median differences	No	No
Saarinen 1999 [20]	Univariable	ANOVA	No	Mean differences	No	No
Jarvinen 2000 [38]	Univariable	ANOVA for repeated measurement	Yes	Mean difference	No	Yes
Rautava 2002 [45]	Univariable	*t*-test,Mann–Whitney	No	Mean difference	No	No
Bottcher 2003 [21]	Univariable	Chi-squared, Fisher’s, Mann–Whitney	No	Median differences	No	No
Oddy 2003 [46]	Multivariable	Chi-squared,multivariable logistic regression	Yes	Mean differences, Odds Ratios	No	No
Savilahti 2005 [22]	Multivariable	Independent samples *t*-test, multivariable logistic regression	Yes	Mean differences, Odds Ratios	No	No
Rigotti 2006 [23]	Univariable	Mann–Whitney, independent samples, *t*-test	No	Median differences	No	No
Snijders 2006 [47]	Multivariable	Multivariable logistic regression	Yes	Odds Ratios	No	No
Bottcher 2008 [24]	Multivariable	Logistic regression	Yes	Odds ratios	No	No
Huurre 2008 [32]	Univariable	Descriptive	No	Descriptive	No	No
Prescott 2008 [33]	Multivariable	Mann–Whitney, Chi-squared, Fisher’s, logistic regression ▪	No	NR	No	No
Tomicic 2010 [34]	Univariable	Mann–Whitney	No	Median differences	No	No
Pesonen 2011 [25]	Multivariable	Two-tailed unpaired *t*-test	Yes	Mean differences	No	No
Kuitunen 2012 [26]	Univariable	Chi-squared, ANOVA, Mantel–Haenszel	No	Geometric means, Odds ratios	No	No
Soto-Ramírez 2012 [36]	Multivariable	Log-linear regression,generalized estimating equations	Yes	Risk Ratio	No	No
Hogendorf 2013 [39]	Univariable	Mann–Whitney	No	Median differences	No	No
Ismail 2013 [43]	Univariable	Mann–Whitney	Yes	Median differences	No	No
Ochiai 2013[27]	Multivariable	Fisher’s, *t*-test, Mann­Whitney, multivariable logistic regression	Yes	Proportions, Odds Ratios, Median differences	No	No
Orivuori 2013 [48]	Multivariable	Multivariable logistic regression	Yes	Odds Ratios	No	No
Joseph 2014 [40]	Multivariable	Logistic regression	Yes	Odds ratios	No	No
Jepsen 2016 [44]	Multivariable	Cox regression	Yes	Hazard ratios	No	No
Simpson 2016 [28]	Multivariable	Logistic regression	Yes	Odds Ratios	No	No
Soto-Ramírez 2016 [35]	Multivariable	Generalized estimating equations	Yes	Risk Ratio	No	No
Munblit 2017 [29]	Multivariable	Binomial GLmulti, LASSO	Yes	Odds Ratios	No	No
Morita 2018 [31]	Multivariable	Mann–Whitney, multivariable logistic regression	Yes	Odds Ratios, Median differences	No	No
Berdi 2019 [30]	Multivariable	Cox regression	Yes	Hazard ratios	No	No

Abbreviations: LASSO, least absolute shrinkage and selection operator. ^1^ Adjustment for potential confounding factors in the model; ^2^ measure(s) of discrimination (receiver operating characteristic (ROC) curve, area under the curve (AUC) or C-statistic, log-rank, D-statistic); ^3^ sensitivity, specificity, predictive values, net reclassification improvement or cut-off points were reported; ▪ it is unclear whether logistic regression was used for assessment of associations between immunological markers and health outcomes.

## Supplementary Materials

The following are available online at https://www.mdpi.com/2072-6643/11/10/2416/s1, Supplemental material: Search strategy.
