# Supplementary material for: Statistical Approaches in the Studies Assessing Associations between Human Milk Immune Composition and Allergic Diseases: A Scoping Review
Source: Nutrients. 2019 Oct 10;11(10):2416. doi: 10.3390/nu11102416 (PMC6836171; doi:10.3390/nu11102416)
Supplement: Supplementary file 1 [file nutrients-11-02416-s001.pdf]

Supplemental material: Search strategy

MEDLINE via Ovid

Ovid MEDLINE(R) and Epub Ahead of Print, In-Process & Other Non-Indexed Citations, Daily and Versions(R) <1946 to December 06, 2018>

1 [Breast milk concept]

2 exp Colostrum/

3 exp Breast Feeding/

4 exp Lactation/

5 Milk, human/

6 (breastmilk or breast-milk or mothersmilk or mothers-milk or ((breast or human? or maternal or mother? or woman) adj2 milk)).ti,ab,kw.

7 (breastfe* or breast-fe* or (breast adj2 (feed* or fed))).ti,ab,kw.

8 (colostrum* or colostral* or colostrium or foremilk or fore-milk or hindmilk or hind-milk).ti,ab,kw.

9 (lactation or breast-secretion* or milk-release or ((breast or milk) adj2 (secretion* or release))).ti,ab,kw.

10 or/2-9

11 [TGF-alpha concept]

12 exp Transforming Growth Factors/

13 Transforming Growth Factor alpha/

14 (TGFa* or TGF-a or TGF-alpha or transforming-growth-factor*).ti,ab,kw.

15 or/12-14

16 [TGF-beta concept]

17 exp TGF-beta Superfamily Proteins/

18 exp Transforming Growth Factor beta/

19 (TGFb* or TGF-b or TGF-beta or bone-morphogenetic-protein? or growth-differentiation-factor? or bone-morphogenic-protein? or BMP?? or BDA? or SSFSC or GDF? or Vgr? or BDA?? or CDMP? LAP? or OS5 or OS-5 or SYM?? or Syn??).ti,ab,kw.

20 or/17-19

21 [C chemokine consept]

22 exp Chemokines/

23 exp Chemotactic Factors/

24 Chemokines, C/

25 (chemokine* or chemotactic-cytokine? or chemotactic-factor? or ATAC or SCM-1* or SCM1* or lymphotactin?).ti,ab,kw.

26 or/22-25

27 [CC chemokine concept]

28 exp Chemokines, CC/

29 (CCL* or I309 or I-309 or TCA? or t-cell-activation or MCP? or monocyte-chemoattractant-protein? or MIP?? or macrophage-inflammatory-protein? or RANTES or C10 or MRP? or MARC or CCF?? or eotaxin or NCC? or Ckb?? or HCC1 or HCC-1 or MCIF or leucotactin? or LEC or LMC or TARC or ABCD? or dendrokine or PARC or DCCK? or DC-CK? or AMAC? or ELC or exodus? or LARC or SLC or secondary-lymphoid-tissue-chemokine? or 6Ckine or

6-Ckine or 6-C-kine or 6C-kine or MDC or MPIF? or eotaxin? or TECK or IMAC or TSC? or CTACK or ILC or PESKY or eskine or skinshine or MEC or LD##).ti,ab,kw.

30 or/28-29

31 [CXC chemokine concept]

32 exp Chemokines, CXC/

33 (CXCL* or NAP1 or NAP2 or NAP-1 or NAP-2 or KC or Gro-b* or PF-4 or PF4 or platelet-factor? or ENA?? or neutrophil-activating-peptide? or GCP? or granulocyte-chemotactic-protein? or CTAPIII or b-Ta or beta-Ta or PEP or interleukin? or MDNCF or GCP? or granulocyte-chemotactic-protein? or MDNCF or gamma-interferon or interferon-gamma or GRG?? or IP10 or IP-10 or I-TAC or bR1 or b-R1 or beta-R1 or beta-R-1 or SDF? or stromal-cell-derived-factor? or PBSF or pre-B-cell-growth-stimulating-factor? or BLC or b-lymphocyte-chemoattractant? or BCA? or BRAK or bolekine or WECHE or lungkine or SRPSOX or DMC or VCC?).ti,ab,kw.

34 or/32-33

35 [CX3C chemokine concept]

36 exp Chemokines, CX3C/

37 (CX3C* or neurotactin or fractalkine).ti,ab,kw.

38 or/36-37

39 [Neurotrophin concept]

40 exp Nerve growth factors/

41 (neurotrophin* or BDNF or neurotropic-factor* or ANON? or BULN? or CNTF or NGF or HSAN? or NGFB or nerve-growth-factor? or NT3 or NT-3 or NTF? or HDNF or NGF? or GDNF).ti,ab,kw.

42 or/40-41

43 [Growth factor concept]

44 exp EGF Family of Proteins/

45 Epidermal Growth Factor/

46 exp Fibroblast Growth Factors/

47 exp Somatomedins/

48 exp Platelet-Derived Growth Factor/

49 exp Vascular Endothelial Growth Factors/

50 Fibroblast Growth Factor 7/

51 (growth-factor* or EGF or HOMG? or URG or ?FGF? or IGF?? or MGF or HBEGF or EGF or HGF or HMGF??? or KGF or PDGF or VEGF or MDGF).ti,ab,kw.

52 or/44-51

53 [Haemopoietic cytokine concept]

54 exp Hematopoietic Cell Growth Factors/

55 (h?emopoietic-cytokine* or EPO or erythropoietin? or MVCD? or ECYT? or DBAL or THPO or MGDF or TPO or thrombopoietin?).ti,ab,kw.

56 or/54-55

57 [Colony-stimulating factor concept]

58 exp Colony-Stimulating Factors/

59 (colony-stimulating-factor* or G-CSF or M-CSF or GM-CSF or GCSF or MCSF or CSF-1 or CSF-2 or CSG-3 or CSF1 or CSF2 or CSF3).ti,ab,kw.

60 or/58-59

61 [Interferon concept]

62 exp Interferons/

63 (interferon? or IFN?).ti,ab,kw.

64 or/62-63
65 [Interleukin concept]
66 exp Interleukins/
67 (interleukin* or IL-1 or IL-2 or IL-3 or IL-4 or IL-5 or IL-6 or IL-7 or IL-8 or IL-9 or IL-1#? or IL-2#? or IL1?? or IL2?? or IL1a* or IL1b* or hematopoietin or catabolin or t-cell-growth-factor or multipotential-CSF or MCGF or BSF? or BCDF? or IFN?? or CSIF or AGIF or NK-cell-stimulatory-factor or P600 or P-600 or LCF or CTLA? or interferon-gamma-inducing-factor?).ti,ab,kw.
68 or/66-67
69 [Cytokine concept]
70 exp Cytokines/
71 (TNF* or tumor-necrosis-factor? or cachectin? or LT-a* or LTalpha or TSLP or SCF or stem-cell-factor? or c-kit or SCGB3A1 or secretoglobin-family-3A-member? or HIN?).ti,ab,kw.
72 or/70-71
73 [Placental growth factor concept]
74 Placenta Growth Factor/
75 (PGF or placental-growth-factor?).ti,ab,kw.
76 or/74-75
77 [IL-6 family concept]
78 Leukemia Inhibitory Factor/
79 (LIF or leukemia-inhibitory-factor or LIF or leukemia-inhibitory-factor or CDF or HILDA or MLPLI or interleukin-6-family-cytokine?).ti,ab,kw.
80 or/78-79
81 [Macrophage migration inhibitory factor concept]
82 Macrophage Migration-Inhibitory Factors/
83 (macrophage-migration-inhibitory-factor? or macrophage-inhibitory-factor? or MIF or MMIF).ti,ab,kw.
84 or/82-83
85 [Osteopontin concept]
86 Osteopontin/
87 (OPN or osteopontin or SPP1 or secreted-phosphoprotein? or BNSP).ti,ab,kw.
88 or/86-87
89 [Oncostatin M concept]
90 Oncostatin M/
91 (OSM or oncostatin).ti,ab,kw.
92 or/90-91
93 [Immunoglobulin concept]
94 exp Immunoglobulins/
95 exp Immunoglobulin Isotypes/
96 (immunoglobulin?? or Ig?? slg??).ti,ab,kw.
97 or/94-96
98 [All signaling peptides/proteins concept]
99 or/15,20,26,30,34,38,42,52,56,60,64,68,72,75,80,84,88,92,97
100 [Animal studies only concept]
101 animals/ not (animals/ and humans/)
102 (10 and 99) not 101

Embase via Ovid

Embase <1974 to 2018 December 07>

1 [Breast milk concept]

2 exp Colostrum/

3 exp Breast Feeding/

4 Lactation/

5 Breast milk/

6 (breastmilk or breast-milk or mothersmilk or mothers-milk or ((breast or human? or maternal or mother? or woman) adj2 milk)).ti,ab,kw.

7 (breastfe* or breast-fe* or (breast adj2 (feed* or fed))).ti,ab,kw.

8 (colostrum* or colostral* or colostrium or foremilk or fore-milk or hindmilk or hind-milk).ti,ab,kw.

9 (lactation or breast-secretion* or milk-release or ((breast or milk) adj2 (secretion* or release))).ti,ab,kw.

10 or/2-9

11 [TGF-alpha concept]

12 exp Transforming growth factor/

13 Transforming Growth Factor alpha/

14 (TGFa* or TGF-a or TGF-alpha or transforming-growth-factor*).ti,ab,kw.

15 or/12-14

16 [TGF-beta concept]

17 Transforming growth factor beta/

18 (TGFb* or TGF-b or TGF-beta or bone-morphogenetic-protein? or growth-differentiation-factor? or bone-morphogenic-protein? or BMP?? or BDA? or SSFSC or GDF? or Vgr? or BDA?? or CDMP? LAP? or OS5 or OS-5 or SYM?? or Syn??).ti,ab,kw.

19 or/17-18

20 [C chemokine consept]

21 exp Chemokine/

22 Gamma chemokine/

23 (chemokine* or chemotactic-cytokine? or chemotactic-factor? or ATAC or SCM-1* or SCM1* or lymphotactin?).ti,ab,kw.

24 or/21-23

25 [CC chemokine concept]

26 Beta chemokine/

27 (CCL* or I309 or I-309 or TCA? or t-cell-activation or MCP? or monocyte-chemoattractant-protein? or MIP?? or macrophage-inflammatory-protein? or RANTES or C10 or MRP? or MARC or CCF?? or eotaxin or NCC? or Ckb?? or HCC1 or HCC-1 or MCIF or leucotactin? or LEC or LMC or TARC or ABCD? or dendrokine or PARC or DCCK? or DC-CK? or AMAC? or ELC or exodus? or LARC or SLC or secondary-lymphoid-tissue-chemokine? or 6Ckine or 6-Ckine or 6-C-kine or 6C-kine or MDC or MPIF? or eotaxin? or TECK or IMAC or TSC? or CTACK or ILC or PESKY or eskine or skinshine or MEC or LD##).ti,ab,kw.

28 or/26-27

29 [CXC chemokine concept]

30 alpha chemokine/

31 (CXCL* or NAP1 or NAP2 or NAP-1 or NAP-2 or KC or Gro-b* or PF-4 or PF4 or platelet-factor? or ENA?? or neutrophil-activating-peptide? or GCP? or granulocyte-chemotactic-

protein? or CTAPIII or b-Ta or beta-Ta or PEP or interleukin? or MDNCF or GCP? or granulocyte-chemotactic-protein? or MDNCF or gamma-interferon or interferon-gamma or GRG?? or IP10 or IP-10 or I-TAC or bR1 or b-R1 or beta-R1 or beta-R-1 or SDF? or stromal-cell-derived-factor? or PBSF or pre-B-cell-growth-stimulating-factor? or BLC or b-lymphocyte-chemoattractant? or BCA? or BRAK or bolekine or WECHE or lungkine or SRPSOX or DMC or VCC?).ti,ab,kw.

32 or/30-31

33 [CX3C chemokine concept]

34 CX3C chemokine/

35 (CX3C* or neurotactin or fractalkine).ti,ab,kw.

36 or/34-35

37 [Neurotrophin concept]

38 Neurotropic agent/

39 (neurotrophin* or BDNF or neurotropic-factor* or ANON? or BULN? or CNTF or NGF or HSAN? or NGFB or nerve-growth-factor? or NT3 or NT-3 or NTF? or HDNF or NGF? or GDNF).ti,ab,kw.

40 or/38-39

41 [Growth factor concept]

42 exp Growth factor/

43 epidermal growth factor derivative/

44 fibroblast growth factor/

45 exp somatomedin/

46 Platelet derived growth factor/

47 Endothelial cell growth factor/

48 Heparin binding epidermal growth factor/

49 Keratinocyte growth factor/

50 (growth-factor* or EGF or HOMG? or URG or ?FGF? or IGF?? or MGF or HBEGF or EGF or HGF or HMGF??? or KGF or PDGF or VEGF or MDGF).ti,ab,kw.

51 or/42-50

52 [Haemopoietic cytokine concept]

53 Erythropoietin/

54 Thrombopoietin/

55 (h?emopoietic-cytokine* or EPO or erythropoietin? or MVCD? or ECYT? or DBAL or THPO or MGDF or TPO or thrombopoietin?).ti,ab,kw.

56 or/53-55

57 [Colony-stimulating factor concept]

58 Colony stimulating factor/

59 (colony-stimulating-factor* or G-CSF or M-CSF or GM-CSF or GCSF or MCSF or CSF-1 or CSF-2 or CSG-3 or CSF1 or CSF2 or CSF3).ti,ab,kw.

60 or/58-59

61 [Interferon concept]

62 exp Interferon/

63 (interferon? or IFN?).ti,ab,kw.

64 or/62-63

65 [Interleukin concept]

66 Interleukin derivative/

67 (interleukin* or IL-1 or IL-2 or IL-3 or IL-4 or IL-5 or IL-6 or IL-7 or IL-8 or IL-9 or IL-1#? or IL-2#? or IL1?? or IL2?? or IL1a* or IL1b* or hematopoietin or catabolin or t-cell-growth-factor or

multipotential-CSF or MCGF or BSF? or BCDF? or IFN?? or CSIF or AGIF or NK-cell-stimulatory-factor or P600 or P-600 or LCF or CTLA? or interferon-gamma-inducing-factor?).ti,ab,kw.
68 or/66-67
69 [Cytokine concept]
70 exp Cytokine/
71 (TNF* or tumor-necrosis-factor? or cachectin? or LT-a* or LTalpha or TSLP or SCF or stem-cell-factor? or c-kit or SCGB3A1 or secretoglobin-family-3A-member? or HIN?).ti,ab,kw.
72 or/70-71
73 [Placental growth factor concept]
74 Placental Growth Factor/
75 (PGF or placental-growth-factor?).ti,ab,kw.
76 or/74-75
77 [IL-6 family concept]
78 Leukemia Inhibitory Factor/
79 (LIF or leukemia-inhibitory-factor or LIF or leukemia-inhibitory-factor or CDF or HILDA or MLPLI or interleukin-6-family-cytokine?).ti,ab,kw.
80 or/78-79
81 [Macrophage migration inhibitory factor concept]
82 Macrophage migration inhibition factor/
83 (macrophage-migration-inhibitory-factor? or macrophage-inhibitory-factor? or MIF or MMIF).ti,ab,kw.
84 or/82-83
85 [Osteopontin concept]
86 Osteopontin/
87 (OPN or osteopontin or SPP1 or secreted-phosphoprotein? or BNSP).ti,ab,kw.
88 or/86-87
89 [Oncostatin M concept]
90 Oncostatin M/
91 (OSM or oncostatin).ti,ab,kw.
92 or/90-91
93 [Immunoglobulin concept]
94 exp immunoglobulin/
95 (immunoglobulin?? or Ig?? slg??).ti,ab,kw.
96 or/94-95
97 [All signaling peptides/proteins concept]
98 or/15,19,24,28,32,36,40,51,56,60,64,68,72,76,80,84,88,92,96
99 [Human studies concept]
100 animal/ not (animal/ and human/)
101 (10 and 98) not 100
